# The Oncogenic Role of CENPA in Hepatocellular Carcinoma Development: Evidence from Bioinformatic Analysis

**DOI:** 10.1155/2020/3040839

**Published:** 2020-04-08

**Authors:** Yuan Zhang, Lei Yang, Jia Shi, Yunfei Lu, Xiaorong Chen, Zongguo Yang

**Affiliations:** ^1^Department of Integrative Medicine, Shanghai Public Health Clinical Center, Fudan University, Shanghai 201508, China; ^2^Department of Acupuncture and Moxibustion, Dongzhimen Hospital, Beijing University of Chinese Medicine, Beijing 100007, China

## Abstract

**Objective:**

This study is aimed at investigating the predictive value of CENPA in hepatocellular carcinoma (HCC) development.

**Methods:**

Using integrated bioinformatic analysis, we evaluated the CENPA mRNA expression in tumor and adjacent tissues and correlated it with HCC survival and clinicopathological features. A Cox regression hazard model was also performed.

**Results:**

CENPA mRNA was significantly upregulated in tumor tissues compared with that in adjacent tissues, which were validated in The Cancer Genome Atlas (TCGA) and Gene Expression Omnibus (GEO) series (all *P* < 0.01). In the Kaplan-Meier plotter platform, the high level of CENPA mRNA was significantly correlated with overall survival (OS), disease-free survival (DFS), recurrence-free survival (RFS), and progression-free survival (PFS) in HCC patients (all log rank *P* < 0.01). For validation in GSE14520 and pan-TCGA dataset, HCC patients with CNEPA mRNA overexpression had poor OS compared with those with low CENPA mRNA (log rank *P* = 0.025 and *P* < 0.0001, respectively), and those with high CENPA had poor DFS in TCGA (log rank *P* = 0.0001). Additionally, CENPA mRNA were upregulated in HCC patients with alpha-fetoprotein (AFP) elevation, advanced TNM stage, larger tumor size, advanced AJCC stage, advanced pathology grade, and vascular invasion (all *P* < 0.05). A Cox regression model including CENPA, OIP5, and AURKB could predict OS in HCC patients effectively (AUC = 0.683).

**Conclusion:**

Overexpressed in tumors, CENPA might be an oncogenic factor in the development of HCC patients.

## 1. Introduction

As a histone H3 variant of centromeric nucleosomes, centromere protein A (CENPA) is required to guarantee kinetochore for proper chromosome segregation assembly [1, 2]. Resulting from artificial overexpression, CENPA mislocalization could potentially generate ectopic kinetochores or weaken native kinetochores, leading to aberrant chromosomal segregation and instable genome [3–5]. An aberrant expression of CENPA and defects in CENPA function result in disrupted genome integrity, abnormal cell division, and thereby cancer [6–8]. Previous reports indicated that nonphysiological incorporation of CENPA may promote human tumorigenesis [9–11].

Previous publications revealed that CENPA was aberrantly overexpressed in hepatocellular carcinoma (HCC) tumor tissues. CENPA promoted HCC cell proliferation both in vitro and in vivo. siRNA-induced CENPA depletion blocked cell cycle progression and assisted apoptosis in HCC cells via numerous genes implicated in cell cycle control and apoptosis. CENPA overexpression was correlated with HBsAg positive status, advanced histological grade, high Ki-67 index, and P53 immunopositivity [12, 13]. However, the clinical predictive value of CENPA in HCC prognosis has not been well illustrated.

This study aimed at investigating CENPA expression in HCC tissues and correlating it with HCC survival and clinicopathological characteristics, in the attempt to validate the oncogenic roles of CENPA and evaluate its predictive value in HCC aggressiveness.

## 2. Materials and Methods

### 2.1. Microarray Data

The microarray profiles including GSE36376, GSE60502, GSE74656, and GSE77314 were obtained from Gene Expression Omnibus (GEO, https://www.ncbi.nlm.nih.gov/geo/). GSE14520 [14, 15] was used to validate the CENPA mRNA expression and its relationship with HCC outcomes. The details of these GEO series were summarized in Table 1. CENPA protein expression detected by an immunohistochemical assay was investigated in the Human Protein Atlas (HPA, https://www.proteinatlas.org/) database [16–18].

Expression and clinical data of liver hepatocellular carcinoma in The Cancer Genome Atlas (TCGA) PanCancer Altas was downloaded from cBioPortal for cancer genomics (http://www.cbioportal.org/) [19, 20].

### 2.2. Survival Analysis

Survival analysis of CENPA in HCC patients was conducted in a Kaplan-Meier plotter [21, 22]. The database is processed by a PostgreSQL server, which integrates both gene expression and clinical data. Patient samples were divided into two groups by a median cutoff of CENPA (RNAseq ID: 1058) to analyze the prognostic value. Two patient groups were compared by the Kaplan-Meier survival plot and the hazard ratio (HR) with 95% confidence intervals (CI), and log rank *P* value was calculated.

Survival validation of CENPA in GSE14520 and TCGA were conducted in Graphpad Prism v8.0 (GraphPad Software, CA, US).

### 2.3. Protein-Protein-Interaction Analysis

Protein-protein interaction analysis of CENPA was performed in online database STRING v11.0 (https://string-db.org/). The minimum required interaction score of parameters interacted with CENPA was set as the highest confidence (≥0.9).

### 2.4. Cox Proportional Hazard Regression Model Establishment

Using the R program, the edgeR package [23] was used for identifying differentially expressed genes (DEGs) in HCC tumor and adjacent tissues, the survival package (https://cran.r-project.org/web/packages/survival/index.html) was used for conducting univariate and multivariate Cox regression analyses, and then, the Cox regression formula was calculated. According to this formula, HCC patients were divided into two groups: the high-risk group and the low-risk group. Survival analysis between these two groups was also performed by survival package. survivalROC (https://cran.r-project.org/web/packages/survivalROC/index.html) was used to perform a receiver operating characteristic (ROC) curve of the model for the prediction of HCC overall survival (OS). pheatmap package (https://cran.r-project.org/web/packages/pheatmap/index.html) was used to summarize the gene expression of parameters in the Cox regression formula.

### 2.5. Statistical Analysis

Graphpad Prism v8.0 (GraphPad Software, CA, US) was used. Student's *t*-test or Mann-Whiney *U* test was performed to analyze the differences of gene expression. Kaplan-Meier survival analysis was performed in GSE14520 and TCGA datasets. A two-tailed *P* < 0.05 was considered significant.

## 3. Results

### 3.1. CENPA Expression

**Figure 1:**
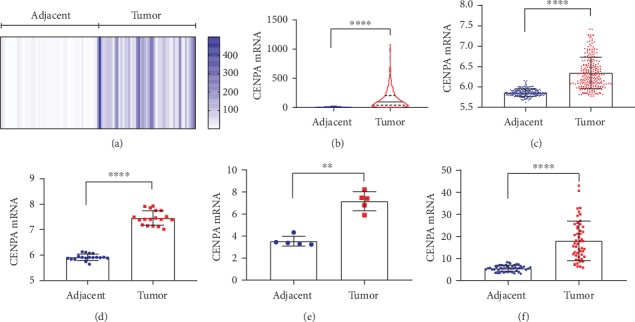
CENPA mRNA expression between tumor and adjacent tissues in HCC patients. CENPA mRNA was significantly upregulated in tumor tissues in 50 paired tumor and adjacent tissues from (a) TCGA profile, (b) entire TCGA profile (*P* < 0.0001), (c) GSE36376 series (*P* < 0.0001), (d) GSE60502 series (*P* < 0.0001), (e) GSE74656 series (*P* < 0.01), and (f) GSE77314 series (*P* < 0.0001).

In TCGA, we obtained 50 paired tumor and nontumor tissues of HCC patients; the heatmap of the CENPA mRNA expression was shown in Figure 1(a), which indicated that CENPA mRNA was apparently upregulated in HCC tumors. When all microarray data of HCC patients in TCGA was included, CENPA mRNA was significantly overexpressed in tumor tissues compared with adjacent tissues (*P* < 0.0001, Figure 1(b)). Consistently, CENPA mRNA were also significantly elevated in HCC tumors compared with those in nontumors in GEO series including GSE36376, GSE60502, GSE74656, and GSE77314 (all *P* < 0.01, Figures 1(c)–1(f)).

**Figure 2:**
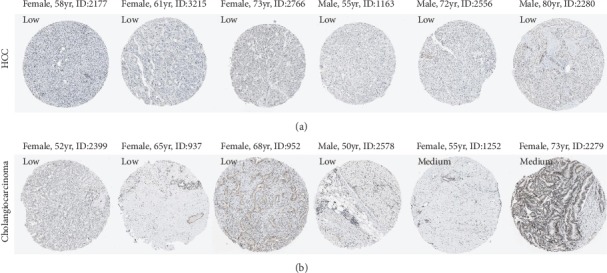
CENPA protein levels in HCC and cholangiocarcinoma detected by immunohistochemical assay in HPA database. (a) 6 HCC patients had low CENPA staining, (b) 4 cholangiocarcinoma patients had low CENPA staining, and 2 had medium staining.

In HPA database, we identified the CENPA protein expression in liver cancer. As shown in Figure 2, 6 HCC patients had low CENPA staining (Figure 2(a)), and 4 cholangiocarcinoma patients had low CENPA staining and 2 had medium staining (Figure 2(b)). Unfortunately, the CENPA protein expression in normal liver tissues was not available.

### 3.2. Associations between CENPA and HCC Survival

**Figure 3:**
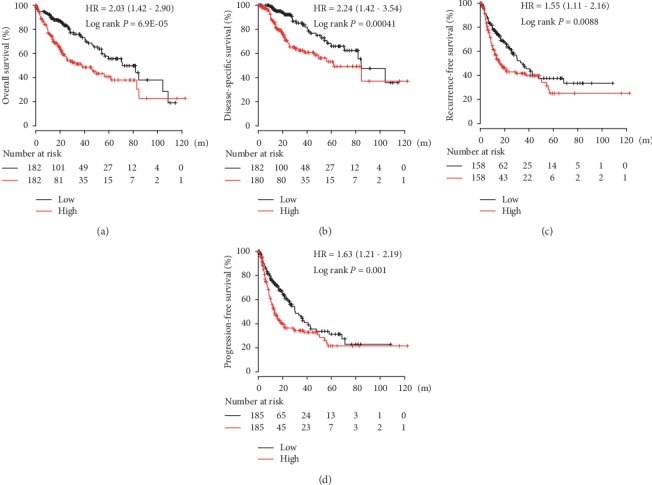
(a) Overall survival, (b) disease-free survival, (c) recurrence-free survival, and (d) progression-free survival in hepatocellular carcinoma (HCC) patients grouped by CENPA mRNA median cutoff in Kaplan-Meier plotter. HCC patients with high CENPA mRNA levels in tumor tissues had worse overall survival (log rank *P* = 6.9*E* − 05) (a), disease-free survival (log rank *P* = 0.00041) (b), recurrence-free survival (log rank *P* = 0.0088) (c), and progression-free survival (log rank *P* = 0.001) (d).

In the Kaplan-Meier plotter platform, CENPA mRNA overexpression was significantly correlated with overall survival (OS), disease-specific survival (DSS), recurrence-free survival (RFS), and progression-free survival (PFS) (HR = 2.03, *P* = 6.9*E* − 05; HR = 2.24, *P* = 0.0041; HR = 1.55, *P* = 0.0088; and HR = 1.63, *P* = 0.001, respectively, Figure 3).

### 3.3. Validation of CENPA in GSE14520 and TCGA

**Figure 4:**
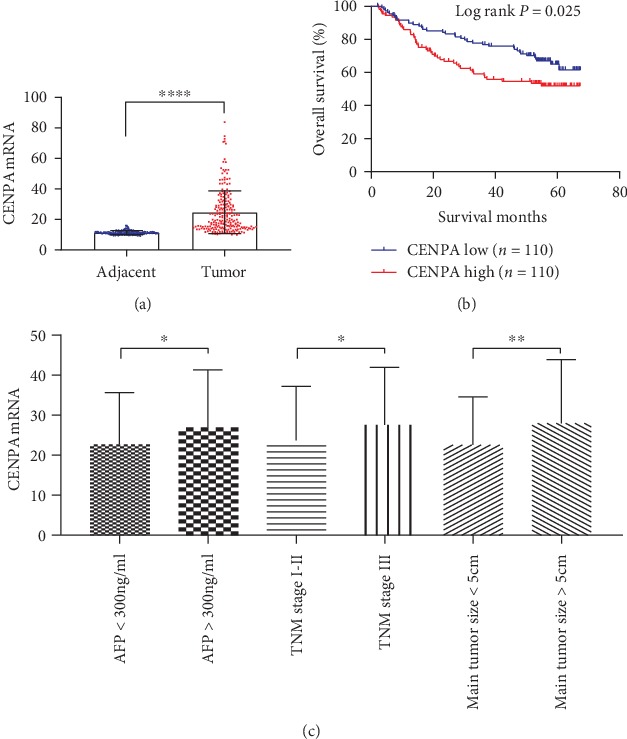
In GSE14520, (a) CENPA mRNA was significantly overexpressed in tumor tissues compared with that in adjacent tissues (*P* < 0.0001); (b) HCC patients with high CENPA mRNA had poorer overall survival compared to those with low CENPA mRNA (log rank *P* = 0.025); and (c) CENPA mRNA was significantly higher in HCC patients with AFP > 300 ng/ml, TNM stage III, and/or main tumor size > 5 cm (all *P* < 0.05).

For validation, we included GSE14520 and TCGA datasets. In GSE14520, CENPA mRNA was significantly upregulated in tumor tissues compared to adjacent tissues (*P* < 0.0001, Figure 4(a)). High CENPA mRNA in tumors is significantly associated with poor OS in HCC patients (*P* = 0.025, Figure 4(b)). In addition, CENPA mRNA overexpression was correlated with AFP elevation, advanced TNM stage, and larger tumor size (all *P* < 0.05, Figure 4(c)).

**Figure 5:**
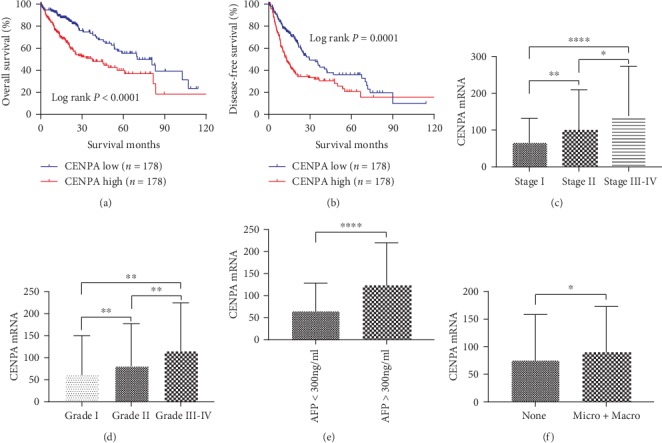
In TCGA dataset, HCC patients with high CENPA mRNA levels had (a) worse overall survival (log rank *P* < 0.0001) and (b) disease-free survival (log rank *P* = 0.0001) compared to those with low CENPA mRNA; CENPA mRNA was significantly increased in HCC patients with (c) advanced AJCC stage (all *P* < 0.05), (d) advanced pathology grade (all *P* < 0.05), (e) AFP > 300 ng/ml (*P* < 0.0001), and (f) vascular invasion (*P* < 0.05).

In the TCGA PanCancer Altas, CENPA mRNA upregulation was negatively associated with OS and DFS in HCC patients (*P* < 0.0001 and *P* = 0.0001, respectively, Figures 5(a) and 5(b)). Also, CENPA mRNA was increased in advanced AJCC stage (all *P* < 0.05, Figure 5(c)) and high pathology grade (all *P* < 0.01, Figure 5(d)). Moreover, CENPA mRNA was significantly elevated in HCC patients with a high AFP level and vascular invasion (*P* < 0.0001 and *P* < 0.05, respectively, Figures 5(e) and 5(f)).

### 3.4. Cox Regression Hazard Model Establishment

**Figure 6:**
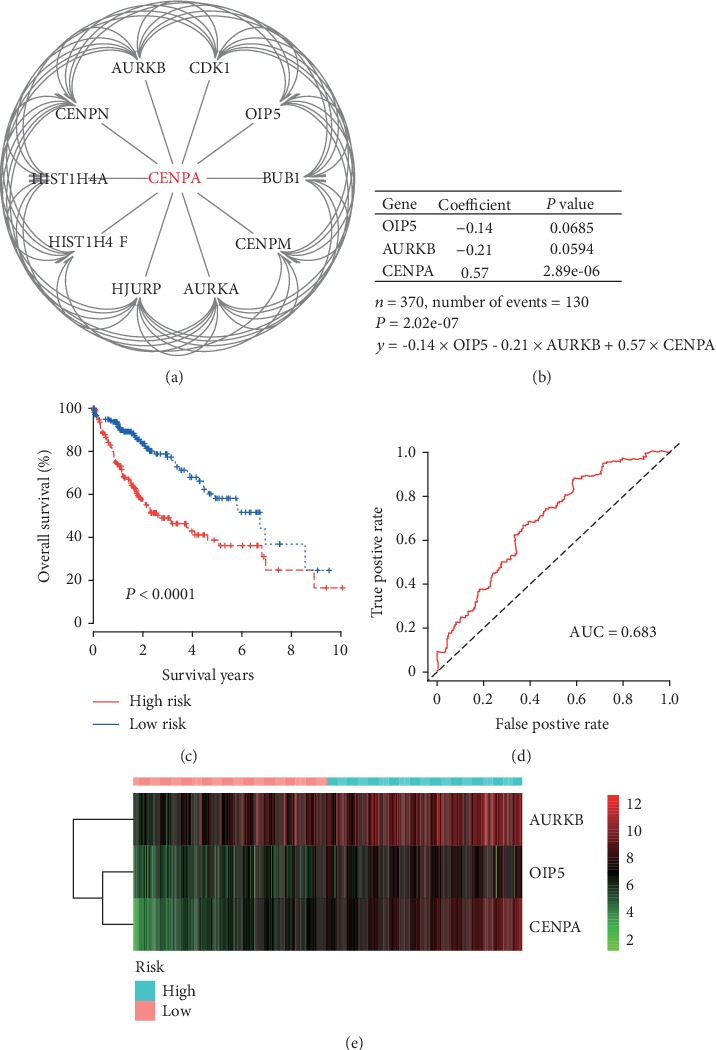
(a) Protein-protein interaction of CENPA in STRING; (b) multivariate Cox regression hazard model of CENPA interactive genes; (c) HCC patients with high risk in the Cox regression model had poorer overall survival than those with low risk (log rank *P* < 0.0001); (d) ROC curve of Cox regression model for overall survival in HCC patients; and (e) heatmap of genes included in the Cox regression model including CENPA, OIP5, and AURKB by risk scores.

Using STRING online service, 10 genes including AURKB, CENPN, HIST1H4A, HIST1H4F, HJURP, AURKA, CENPM, BUB1, and OIP5 were interacted with CENPA (Figure 6(a)). In R program, univariate and multivariate Cox regression included 3 genes (CENPA, AURKB, and OIP5) in the Cox proportional hazard regression model, with a formula as *y* = −0.14 × OIP5–0.21 × AURKB + 0.57 × CENPA (Figure 6(b)). According to the formula, HCC patients were divided into the high-risk group and the low-risk group, with a median risk score. As shown in Figure 5(c), HCC patients with high risk had poor OS compared with low-risk patients (*P* < 0.0001, Figure 6(c)). To identify the accuracy of this model for the prediction of OS in HCC patients, the ROC curve with an area under the curve (AUC) equal to 0.683 was summarized in Figure 6(d). The heatmap of CENPA, OIP5, and AURKB expression in high-risk and low-risk HCC patients was summarized in Figure 6(e).

## 4. Discussion

Altered expression of CENPA was frequently observed in many types pf human malignancies including lung cancer, colorectal cancer, breast cancer, and HCC [11, 24–27] and implicated in cell cycle regulation, cell survival, and genetic stability [11, 12, 28, 29]. Our results from several datasets revealed that CENPA mRNA was significantly upregulated in HCC tumors, which is consistent with previous reports [26, 28]. Using immunohistochemical analysis, Li et al. showed that the positive rate of CENPA in HCC samples was significantly higher than nonneoplastic liver tissues [26]. Considering both our results and the previous reports, we assumed that CENPA should be an oncogene in HCC progression.

The prognostic value of CENPA in human cancers was partly illustrated [11]. CENPA was an independent prognostic factor for lung adenocarcinoma, primary osteosarcoma, and epithelial ovarian cancer [24, 30–32]. High CENPA was significantly associated with advanced pathological grade, pT status, pN status, pleural invasion, high Ki-67 expression, and p53 positivity in lung adenocarcinoma [24, 32]. Similarly, CENPA upregulation was associated with tumor size, cancer recurrence, lung metastasis, high Ki-67 expression, and p53 positivity in primary osteosarcoma [30]. Additionally, CENPA elevation was significantly correlated with advanced pathology grade, tumor stage, and poor survival in human epithelial ovarian cancer [31]. CENPA was an independent predictor for relapse in estrogen receptor- (ER-) positive breast cancer patients not receiving systematic therapy but not in ER-negative patients. And CENPA also was not a prognostic marker of chemotherapy response [10]. Increased CENPA showed weak association with DFS in invasive breast cancer [27].

Unfortunately, few reports have focused on the clinical relationships between CENPA and HCC progression. A report by Li et al. found that CENPA overexpression was associated with advanced histological grade, positive serum HBsAg status, Ki-67 expression, and p53 immunopositivity [12]. Our results revealed that CENPA upregulation was correlated with poor outcomes (OS, DSS, RFS, and PFS) and progressive clinico-pathological features including AFP elevation, advanced tumor stage, vascular invasion, and tumor size in HCC patients, which were validated in both GSE14520 and TCGA datasets. Hence, we supposed that CENPA might play an oncogenic role in HCC progression. Previous basic experiment studies indicated that CENPA knockdown could reduce cell proliferation, block cell cycle at G1 phase, and induce apoptosis in HepG2 cells. Conversely, CENPA overexpression promoted cell growth and reduced apoptosis in HCC cell [12, 28]. Additionally, HBx deletion was frequently observed in HBV-related HCC tissues. CENPA expression was positively associated with HBx mutation in HCC tissues. And HBx mutant increased CENPA mRNA and protein expression remarkably compared to full-length HBx [13].

This report has some limitations. Firstly, the analysis was based on online bioinformatic analysis in transcriptional level, without CENPA protein data. Secondly, we could not perform experimental research for probing potential oncogenic mechanisms of CENPA in HCC development. Thirdly, this study had relatively small samples and performed within limited institute, and there was no follow-up data for our own from available HCC patients. Prospective studies with large sample should be performed to validate the results of this analysis. Even though we considered previous reports, we cautiously drew the hypothesis that CENPA overexpression contributed to unfavorable prognosis in HCC patients.

## Figures and Tables

**Table 1:** Details of GEO series included in this analysis.

GEO series	Contributor(s)	Tumor	Nontumor	Platform
GSE14520	Roessler S et al., 2009	222	212	Affymetrix Human Genome U133A 2.0 Array/Affymetrix HT Human Genome U133A Array
GSE36376	Park CK, 2012	240	193	Illumina HumanHT-12 V4.0 expression beadchip
GSE60502	Kao KJ, 2014	18	18	Affymetrix Human Genome U133A Array
GSE74656	Yin H, 2015	5	5	GeneChip® PrimeView™ Human Gene Expression Array (with external spike-in RNAs)
GSE77314	Hou G et al., 2016	50	50	Illumina Genome Analyzer (Homo sapiens)

## Data Availability

The data used to support the findings of this study are available from the corresponding author upon request.
